# Multi-method study of the implementation of Cognitive Symptom Management and Rehabilitation Training (CogSMART) in real-world settings

**DOI:** 10.1186/s12913-022-08941-z

**Published:** 2022-12-17

**Authors:** Laurie Lindamer, Erin Almklov, James O. E. Pittman, Shuyuan Shi, Jacqueline Maye, Amy Jak, Elizabeth Twamley, Borsika Rabin

**Affiliations:** 1 VA Center of Excellence for Stress and Mental Health, 3350 La Jolla Village Dr., San Diego, CA 92161 USA; 2 Department of Psychiatry, University of California San Diego, 9500 Gilman Dr., La Jolla, CA 92093 USA; 3 Department of Psychology, University of British Columbia, 2136 West Mall, Vancouver, BC V6T 1Z4 Canada; 4 Department of Family Medicine and Public Health, University of California San Diego, 9500 Gilman Dr., La Jolla, CA 92093 USA

**Keywords:** CogSMART, RE-AIM, Dissemination, Implementation, Traumatic Brain Injury (TBI), Neuropsychiatric disorders, Multi method

## Abstract

Cognitive Symptom Management and Rehabilitation Training (CogSMART) and Compensatory Cognitive Training (CCT) are evidence-based compensatory cognitive training interventions that improve cognition in persons with a history of traumatic brain injury or other neuropsychiatric disorders. Despite demonstrated efficacy, use and effectiveness of CogSMART/CCT in real-world settings is not known.

We used a multi-method design to collect and analyze quantitative and qualitative survey data from several domains of the Reach, Effectiveness, Adoption, Implementation, and Maintenance (RE-AIM) framework to gather information about use of CogSMART/CCT in real-world settings from provider and patient perspectives. Surveys were sent to email addresses from persons who registered on the CogSMART website seeking access to training manuals and other resources. Descriptive statistics were generated, and we used Natural Language Processing methods to study the self-report free responses. Using n-gram analysis, we identified the most frequently reported responses.

We found CogSMART/CCT was broadly used in real-world settings and delivered by a variety of providers for several patient groups with high attendance and overall high satisfaction. CogSMART/CCT seemed to be offered in VA- or university-related clinics more than in private practice or rehabilitation centers. The diversity of providers and variety of formats by which to deliver CogSMART/CCT (i.e., individual, group, telehealth) seemed to play a role in its widespread implementation, as did its adaptability. Most providers made adaptations to the intervention that reduced the length or number of sessions. These changes were most likely to be based on client characteristics. The low rates of formal training, however, may have contributed to lower levels of perceived helpfulness among patients.

Reach and Adoption of a cognitive rehabilitation intervention improved by increasing access to the manuals. Attention to characteristics of dissemination and implementation in the design of an intervention may enhance its use in real-world settings. The relevant outcomes, easy access to training manuals, and adaptability of CogSMART/CCT seem to have been important factors in its use in a variety of settings and for several disorders with cognitive impairment. The adoption of CogSMART/CCT by a variety of providers other than neuropsychologists suggests its use may be broadened to other healthcare providers, if adequately trained, to increase access to an intervention with demonstrated efficacy for cognitive rehabilitation for several neuropsychiatric disorders.

## Background

Cognitive rehabilitation has been shown to be effective in increasing cognitive performance and daily functioning for persons with TBI and other neurocognitive disorders [1]. The compensatory cognitive training (CCT) rehabilitation approach employs cognitive techniques and coping strategies, such as problem-solving and use of external aids, to improve cognitive performance and functional capacity [1]. In one such CCT-based intervention, Cognitive Symptom Management and Rehabilitation Training (CogSMART), individuals are taught strategies and habits that specifically target memory, attention, prospective memory, and executive functioning. They learn to make use of internal strategies (e.g., visual imagery, information chunking, acronyms, problem solving, planning) and external aids (e.g., day planners and timers), to augment their own current cognitive resources. In clinical trials, CogSMART training has been shown to improve cognition, functional capacity, post-concussive symptom severity, and quality of life in civilians and Veterans with TBI [2–7], as well as in several other populations of patients with neurocognitive disorders including serious mental illness and psychosis [8–11], co-occurring PTSD and TBI [12], hoarding disorder [13–17], autism [18], and HIV [19].

Despite its demonstrated efficacy, CogSMART/CCT, however, like many other evidence-based practices (EBPs), is not widely implemented in clinical practice. To our knowledge, there is one study reported in the literature that investigated a multi-component cognitive intervention for older adults in a real-world setting. Mao and colleagues (2021) found that this intervention improved cognition compared to preintervention levels when it was delivered in day care and neighborhood centers in Taiwan (Mao, et al. 2021) [20]. The lag in time to translate EBPs into wide use in practice settings is often due to a lack of fit between the intervention, implementation strategies, and the intended contexts, as well as many external environmental factors [21, 22]. The investigation of the use of and adaptations to EPBs in controlled settings is one approach to understanding their dissemination, adoption, implementation, and sustainment. Another approach to understand the uptake of EBPs is to examine their active dissemination in the real-world environment. CogSMART/CCT was designed with several features to enhance uptake, such as targeting the improvement of outcomes that are meaningful to the patients, no-cost access to the training manuals and implementation support materials, and marketing to providers and patients (website, YouTube videos).

To better understand naturalistic dissemination and implementation of CogSMART/CCT in real-world settings, we surveyed providers and patients who downloaded the CogSMART/CCT manuals through the research-related website. We employed the RE-AIM (Reach, Effectiveness, Adoption, Implementation, and Maintenance) framework [23] to evaluate the implementation outcomes of CogSMART in real-world settings.

Applying the pragmatic RE-AIM approach [24], we sought to answer the following questions: Who is intended to benefit from CogSMART/CCT and who participated in the intervention (Reach)? What were the benefits of CogSMART/CCT from the patient and provider perspectives (Effectiveness)? Who delivered CogSMART/CCT and where was it delivered (Adoption)? How consistently was CogSMART/CCT administered and how was it adapted (Implementation)? We did not address maintenance (e.g., how long will a provider use CogSMART/CCT and how long the benefits of it last) in this study.

The purpose of this study was to explore the use of an EBP for cognitive rehabilitation, CogSMART/CCT, in real-world settings to inform its future implementation.

## Method

This study used a multi-method design to collect and analyze quantitative and qualitative survey data to gather information about the use of CogSMART/CCT in real-world settings from provider and patient perspectives.

### Survey development

Using the pragmatic RE-AIM framework, we developed the survey and piloted it with research assistants familiar with the intervention to refine the questions. Most responses were categorical or ordinal, and some Likert scales and free text fields were included. The provider survey was 58 total questions, and the patient version contained 73. The survey was designed to minimize response burden; therefore, questions were presented in a sequential rather than randomized manner, and no completeness or review checks were included. We did not offer incentives for participation; therefore, we attempted to reduce response burden. The survey was re-issued to the same email list with an additional question regarding the perceived overall helpfulness for the providers to compare to patient data yielding a sample of 19 and 16, respectively. Email addresses we examined for duplicates, and, if a second response was detected, we used the data from the used the response that was more complete. Skip logic was employed, and participants could choose not to answer any question.

### Participants and survey administration

This open survey was administered to a convenience sample of nearly 6000 individuals who provided email addresses to access treatment manuals and other intervention-related tools available through the researcher-designed website from January 2014 to June 2020. Links to the survey were included in an email update notifying the recipient of the availability of an updated manual. Participation was anonymous and voluntary. Respondents self-identified their role as provider or patient by selecting the corresponding survey link. The survey was active for from March 2020 to June 2020, and two reminders were sent two and four weeks after the initial invitation. In January 2021 we reissued the survey for three months to increase participation, as well as to collect expanded and standardized information across stakeholders on perceived helpfulness of and satisfaction with CogSMART/CCT, which resulted in different patient sample sizes for some variables. There were no significant differences between current and past users on the intervention characteristics, so the data were combined for analysis. The study was conducted via Qualtrics, and data was stored behind a VA-secured firewall.

Additional data on patients’ perceived helpfulness was obtained from a quality improvement project in the Cognitive Rehabilitation Clinic at VA San Diego Healthcare System (VASDHS) (*n* = 86) with data collected from 2011 to 2016. It included the CogSMART Feedback Form, which consists of a 10-item list of cognitive domains, post-concussive symptoms, psychoeducation, and information about additional services. The perceived treatment helpfulness for each item was rated on a five-point Likert scale (1 = not helpful; 2 = mildly helpful; 3 = moderately helpful; 4 = very helpful; and 5 = extremely helpful). All participants in the clinic were Veterans with TBI participating in CogSMART/CCT. No information on demographic or clinical characteristics was available. Therapists were master’s level staff who received intensive training that consisted of manual review with neuropsychologist, observation of a neuropsychologist delivering the intervention, delivery of the intervention with observation, and then serving as co-therapist with a CogSMART/CCT expert. Despite the lack of patient characteristics, these data provided the opportunity to contrast the use of CogSMART/CCT conditions with lower external and higher internal validity (structured clinical conditions) to those with higher external and lower internal validity (real world settings).

### Data analysis

Quantitative survey data was characterized using descriptive statistics including frequencies, percentages, and graphs. Chi-square tests were also used to assess for statistical differences in perceived helpfulness ratings. All quantitative analyses were conducted using SPSS 27. Qualitative data (i.e., free text survey questions inquiring about barriers and facilitators of CogSMART/CCT as well as “Any other suggestions”) were analyzed with Python using Natural Language Processing methods - n-gram analysis [25] - to identify response categories and frequencies, such as the most frequently reported responses.

### Ethical concerns

The survey was reviewed and approved by the VASDHS IRB (HRD200006). Respondents were told of the voluntary nature of their participation, informed that they could refuse to answer questions, and acknowledgement of consent was required prior to beginning the survey.

## Results

### Dissemination of CogSMART/CCT

Of the 5853 unique email addresses, the most frequent domains were from the VA, educational institutions, or international organizations, other US government agencies, and healthcare-related organizations (*n* = 2652) with the remainder consisting of commercial domain addresses (*n* = 3142) and undetermined (*n* = 50). Respondents included healthcare providers and patients (*n* = 207 and 87 respectively) who were past or current users of CogSMART/CCT.

Providers in real-world settings reported that the most frequent source of information about CogSMART/CCT was from colleagues (46%) with journal articles (20%), other sources (19%) and workshops (15%) less likely. Patients were most likely to hear about CogSMART/CCT from their healthcare providers (54.4%) compared to workshops (18.2%), other sources (18.2%), or journals (4.5%).

### Reach

Because this was a naturalistic study of the dissemination and implementation of CogSMART/CCT, in real-world settings, we did not determine *a priori* the intended recipients of this intervention. We did ascertain, however, the characteristics of 75 patients who voluntarily responded to the internet survey and provided the data on the website; twelve individuals declined to answer the survey questions. See Table 1 for patient demographics.

**Table 1:** Patient Characteristics

Located in the US (*N* = 87)	Yes = 75, 86%
Gender (*N* = 43)	Males = 10, 23%
	Female = 31, 72%
	Nonbinary = 2, 5%
Age (*N* = 40)	M = 49.2, SD = 13.1
Education (*N* = 41)	Completed high school/GED = 1, 2.4%
	Some college/training after high school = 5, 12.2%
	Graduated college with an Associate degree = 5, 12.2%
	Graduated college with a Bachelor degree = 11, 26.8%
	Some postgraduate training = 2, 4.9%
	Post graduate degree = 17, 41.5%
Work Status (*N* = 44)	Receiving disability or similar support = 18, 41%
	Part-time employment = 5, 11.4%
	Full-time employment = 21, 47.7%
Received Care at (*N* = 46)	VA hospital or clinic = 9, 19.6%
	University-affiliated hospital or clinic = 5, 10.9%
	Other hospital or clinic = 8, 17.4%
	Private Practice = 6, 13.0%
	Rehabilitation Facility = 1, 2.2%
	Did CogSMART/CCT on my own = 8, 17.4%
	Other = 9. 19.6%

Patients were referred to CogSMART/CCT most frequently based on provider assessment and clinical judgment and patient goals. Other reasons for referral included diagnosis or participation in an assessment or treatment clinic. Most of the providers reported using TBI-related CogSMART/CCT manuals (67%), with 26% using manuals for psychiatric illnesses and 7% using manuals for mild cognitive impairment.

### Effectiveness

#### Overall satisfaction and perceived helpfulness

Based on the providers who answered this question (*N* = 102), 99% (*N* = 101) would recommend CogSMART/CCT to others, and for the patients who responded (*N* = 19), 95% (*N* = 18) would recommend CogSMART/CCT. There were no significant differences between the patients and providers. Ratings for perceived helpfulness are presented in Table 2.

**Table 2:** Perceived Overall Helpfulness for Providers and Patients

	Not Helpful	Mildly Helpful	Moderately Helpful	Extremely Helpful	Don’t Know
Providers (*N* = 19)	--	21%	53%	21%	5%
Patients (*N* = 16)	6.3%	6.3%	37.5%	37.5%	12.5%

#### Perceived helpfulness of cognitive domains

Perceived helpfulness of strategies taught in the four CogSMART/CCT cognitive domains (prospective memory, attention, learning and memory, and executive functioning) and, in the case of TBI manuals, information provided on post-concussive symptoms, were compared for providers across disciplines, settings, and neurocognitive conditions. The perceived helpfulness of CogSMART/CCT content domains generally had high helpfulness ratings across settings, manuals/conditions (traumatic brain injury, serious mental illness, mild cognitive impairment), and disciplines with no statistically significant differences observed.

**Fig. 1:**
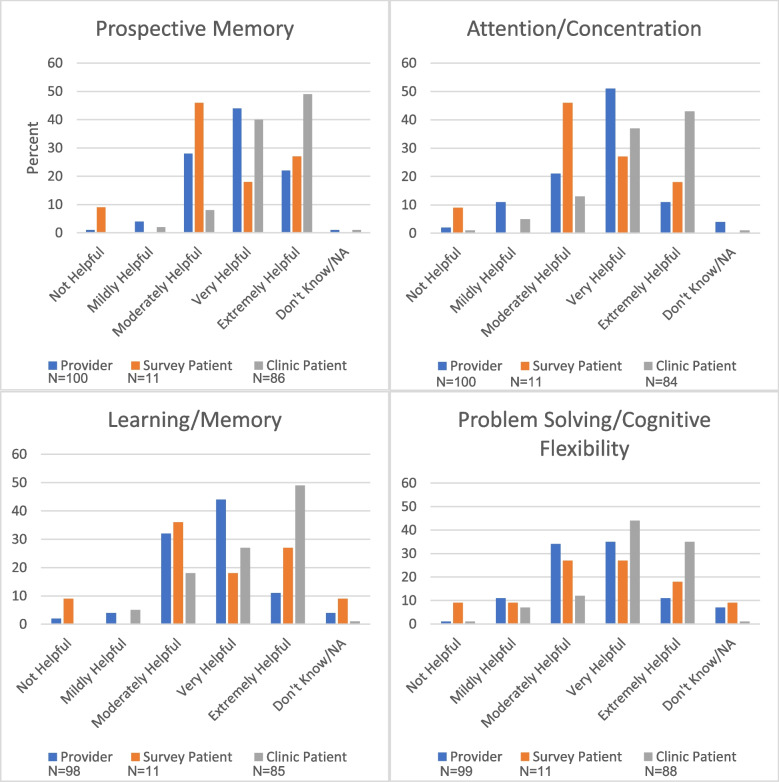
Perceived Helpfulness of CogSMART/CCT Domains by Provider and Patient Groups

Patients seen in the VASDHS Cognitive Rehabilitation Clinic were significantly more likely to rate perceived helpfulness higher for the cognitive domains of prospective memory, learning and memory, and problem solving than both patients and providers who responded to the survey (See Fig. 1). Providers were more likely to rate helpfulness for the attention/concentration domain higher than patients in real world or clinical settings.

### Adoption

Provider characteristics can be seen in Table 3.

**Table 3:** Provider Characteristics

Located in the US (*N* = 181)	Yes = 139, 77%
Gender (*N* = 180)	Male = 33, 18%
	Female = 146, 81%
	Non-binary/Other = 1, 0.5%
Age (*N* = 172)	M = 42.5, SD = 11.4
Years in practice (*N* = 138)	M = 12.4, SD = 9.6
Received Training (*N* = 114)	No = 76, 67%
	Yes = 38, 33%
Profession (*N* = 178)	Psychologist = 38, 21.3%
	Neuropsychologist = 65, 36.5%
	Psychiatrist = 3, 1.7%
	Nurse or Nurse Practitioner 1, 0.5%
	Social Worker = 3. 1.7%
	Speech/Language Pathologist (ST) = 28, 15.7%
	Occupational Therapist (OT) = 16, 9%
	Other = 24, 13.5%
Provided Care at (*N* = 179)	VA Hospital or Clinic = 55, 30.7%
	University Affiliated Hospital or Clinic = 35, 19.6%
	Other Hospital or Clinic = 34, 19%
	Private Practice = 26, 14.5%
	Rehabilitation Facility = 14, 7.8%
	Other = 15, 8.4%
Manual Type	TBI = 139, 67%
	CCT Psychiatric = 53, 26%
	CCT Mild Cognitive Impairment (MCI) = 14, 7%
Therapy Format (*N* = 160)	Group = 47, 29%
	Individual = 80, 50%
	Telehealth = 25, 16%
	Other = 8, 5%

About 67% of providers currently providing CogSMART/CCT reported no formal training in CogSMART/CCT prior to delivering the intervention independent of whether they were neuropsychologists. Of those receiving training, neuropsychologists and psychologists had the highest rates of formal training (50% and 36%) compared to other disciplines (ST (18%) and OT 18%) and other (31%). Those with formal training were more likely to be treating TBI, rather than other neurocognitive disorders, and work in a VA- or university-affiliated hospital. The training of the providers in the Cognitive Rehabilitation Clinic versus the real-world setting differed considerably. All the providers in the clinic setting received intensive training, whereas the majority (67%) of those in the real-world setting received no formal training.

### Implementation

Providers reported that patients attended an average of eight sessions of the 10–12 session intervention (75–80% attendance rate) when CogSMART/CCT was delivered in individual or group format. Patients participating in the intervention using telehealth format attended more than half of the sessions. High attendance rates were further supported by patient responses: 63% reported attending all or most sessions, 26% at least half of the sessions, and only 11% less than half.

Most providers (74%) reported making modifications to CogSMART/CCT. The most frequent adaptations were combining (14%), modifying (17%) or omitting (17%) exercises and shortening the length (15%) or number of the sessions (14%). Most providers based these adaptions on client characteristics (42.5%), such as language or cultural adaptations or reading level, or logistics (31%) followed by provider preference (13.7%) and other reasons (9%). Very few providers made adaptations based on evidence (3%).

Qualitative data was collected from questions inquiring about barriers and facilitators of CogSMART/CCT, as well as from answers to the question “Any other suggestions?” (See Table 4).

**Table 4:** Frequency of Barriers and Facilitators

Barriers	% of total response(*n* = 74)	Facilitators	% of total response (*n* = 70)
**Patient Characteristics**			
• Patient participation (attendance, adherence, and motivation)	30		
• Fit (client expectation/complaint, generalizability/individualization)	16		
• Client characteristics (level of impairment)	15		
**Intervention Characteristics**			
• Time (length of the program, scheduling and coordination, time constraint of sessions)	20	• Manuals (structured, clear, with examples and practices, user-friendly, easy to follow)	39
• Length/density/difficulty of the material for the patient	16	• Helpful/practical strategies/practices/worksheets with explanation of relevant information	23
• Limited knowledge in delivering the program	1	• Therapists and Psychologists	16
• Language	1	• Easily accessible with the online format	10
		• Adaptability to different programs (outpatient, outreach, residential)	--
		• Engaging Videos	4
		• No-cost	3
		• Consultation with the creator	1

Despite overall reports of high attendance and the modifiability of CogSMART/CCT, some providers described barriers related to both patient and intervention characteristics. For example, patient adherence and motivation and the length of the session or intervention were the most frequent patient and intervention characteristics noted as barriers, respectively. Fit of the patient and intervention were also noted as somewhat problematic, and providers suggested that there is a need to further adapt the intervention to patient’s complaints and lower level of functioning. Additional suggestions focused on addressing attention and memory in earlier sessions, and implementing metacognitive strategies, more executive functioning exercises, and more practical applications of cognitive flexibility.

In contrast, the accessibility of the intervention and its pragmatic focus were perceived as facilitators. For example, providers noted the benefit of CogSMART/CCT on patient outcomes, its flexibility to combine with other approaches, and its usefulness for training purposes (“[CogSMART/CCT is] Super useful in building patient confidence, practical and easy to combine with other approaches” and “Helpful as both a stand-alone intervention or blend in with other approaches….”). The quality of the examples and exercises was also noted. Others mentioned CogSMART/CCT’s appropriateness for other populations, such as multiple sclerosis and mild cognitive impairment.

## Discussion

Our study describes the naturalistic dissemination and implementation of CogSMART/CCT in real-world settings using a convenience sample of providers and patients who requested access to CogSMART/CCT training manuals from the website. Data showed that the intervention was broadly disseminated and widely used in real-world settings. CogSMART/CCT was delivered by a variety of providers for several patient groups with overall high attendance and limited, but high satisfaction scores. The low rates of formal training, however, may have contributed to patients’ lower levels of perceived helpfulness among cognitive domains. Data from this sample indicated that CogSMART/CCT is more frequently offered in VA- or university-affiliated clinics than in private practice or rehabilitation centers. Adaptations to CogSMART/CCT included the reduction of the number of exercises or shortening the length or number of sessions, and these changes were most likely to be based on client characteristics. Some providers, however, noted that further improvements of the intervention for patients with lower levels of functioning would be beneficial.

Our results are consistent with the literature showing that attention paid to design characteristics in developing interventions increases their reach [26]. CogSMART/CCT was designed to be accessible, easy to use, and practical. The creation of an intervention-specific website with open access clearly resulted in dissemination of this EBP. CogSMART/CCT’s ease of use and focus on outcomes relevant to patients may have also contributed to the use of CogSMART/CCT by providers and patients. Diffusion, the social influence that facilitate knowledge of, attitudes toward, and use of an intervention, also seemed to broaden the reach of CogSMART/CCT, because the most common method that providers and patients learned of it was from colleagues or healthcare providers respectively [26]. The reach of other EBPs may be enhanced by attending to factors related to dissemination and diffusion early in the design of the intervention.

CogSMART/CCT was designed for several patient groups demonstrating neurocognitive impairment, and our findings of real-world use are consistent with this. Pragmatic trials and implementation studies to capture data RE-AIM outcomes in more representative samples are needed.

Despite its broad dissemination and limited but overall high satisfaction, patients’ perceived helpfulness of CogSMART/CCT may be associated with the training of the provider. Patients treated in the VA Cognitive Rehabilitation Clinic, where all the providers received intensive training, rated perceived helpfulness of several cognitive domains higher than did patients in the real-world settings. In contrast, we observed that training differed among provider types, although these differences were not significant. A critical role in translating EBPs into broad practice involves effective provider training [27]. The fidelity with which EBPs are delivered affects patient outcomes, emphasizing the need for well-trained providers [28]. Future research on the relationship of types and intensity of training in the delivery of CogSMART/CCT is warranted to achieve the best possible patient outcomes with the most cost-effective training modality. Moreover, the delivery of CogSMART/CCT in real world settings by providers other than neuropsychologists, suggests that the use of non-specialty providers may further extend access to evidence-based cognitive training for persons with TBI and other cognitive impairments. The use of task-sharing has been used to increase the provision of mental health care services in rural and low-resource settings [29, 30]. Task-shifting entails reallocating duties, typically from more to less highly trained individuals to make efficient use of these resources, allowing all providers to work at the top of their scope of practice [31]. Effective training with adequate fidelity to EBPs will likely require tailoring the educational materials to the unique characteristics and contexts of the potential group of providers.

Data from the implementation of CogSMART/CCT in real world settings indicated that the intervention was frequently adapted to accommodate the characteristics of the patients, and that this adaptability was a facilitator in its adoption by providers. Current literature on the adaptation of EBPs differentiates between core functions, the underlying mechanisms of change that make an intervention effective, and forms, which are the specific intervention activities [32]. The high satisfaction and perceived helpfulness ratings of CogSMART/CCT by both the providers and patients suggest that the potential mechanisms of change (i.e., psychoeducation, skill development, practice, problem solving, and generalization) were easily identifiable and preserved, while the details of the intervention (i.e., delivery format, exercises, and length and number of sessions) were modified to meet the needs of the patients. Future studies are needed to confirm core functions and forms for CogSMART/CCT, but others have identified four techniques (facilitation by a therapist, cognitive exercise, procedures to develop problem-solving strategies, and procedures to facilitate transfer to real world functioning) in cognitive remediation for persons with schizophrenia [33].

There are several limitations of this study. Most notable is the potential of response bias, given the very low response rate, particularly related to overall satisfaction and perceived helpfulness. The purpose of this study, however, was not hypothesis testing, but rather the exploration of the natural diffusion of CogSMART/CCT and the characterization of its use in real world settings using mixed methods. Several findings from these data suggest future important and actionable research, such as the relationship between training and outcomes, task shifting as an implementation strategy, and understanding of core forms and functions to enable systematic adaptation. More research in these areas, as well as in the match between the intervention, implementation strategies, and environment, is needed.

## Conclusion

Manualized cognitive rehabilitation interventions with known efficacy can have a broad reach and be delivered in a variety of settings when they are available and accessible to healthcare professionals and patients. Attention to characteristics of dissemination and implementation in the design of an intervention may enhance its use in real-world settings. The relevant outcomes, easy access to training manuals, and adaptability of CogSMART/CCT seem to have been important factors to its use in a variety of settings and for several disorders with cognitive impairment. A key finding of our study was to emphasize importance of local adaptation of the intervention to increase the fit to the patient population and context. Adaptations can include changes to the number, length, and content of sessions.” The adoption of CogSMART/CCT by a variety of providers other than neuropsychologists, suggest that its use may be broadened to other healthcare providers, if adequately trained, to increase the access to an intervention that has demonstrated efficacy for several neuropsychiatric disorders.

## Data Availability

The datasets supporting the conclusions of this article are available from the corresponding author on reasonable request.

## Abbreviations

TBI: Traumatic Brain Injury
CogSMART: Cognitive Symptom Management and Rehabilitation Training
CCT: Compensatory Cognitive Training
RE-AIM: Reach, Effectiveness, Adoption, Implementation, and Maintenance framework
PTSD: Post-traumatic Stress Disorder
EBP: Evidence-based Practices
VASDHS: VA San Diego Healthcare System
IRB: Institutional Review Board
MCI: Mild Cognitive Impairment
ST: Speech Therapist
OT: Occupational Therapist
